# Embolism resistance supports the contribution of dry-season precipitation to transpiration in eastern Amazon forests

**DOI:** 10.1073/pnas.2501585122

**Published:** 2025-08-14

**Authors:** Magali F. Nehemy, Caio R. C. Mattos, Rafael S. Oliveira, Marina Hirota, Ying Fan, Monique B. Schlickmann, Deliane Penha, Leandro L. Giacomin, Julliene S. G. M. Silva, Mayda Rocha, Gleicy A. Rodrigues, Jeffrey J. McDonnell

**Affiliations:** ^a^Department of Earth and Environmental Sciences, The University of British Columbia Okanagan, British Columbia V1V 1V7, Canada; ^b^School of the Environment, Trent University, Peterborough K9J 0G2, Canada; ^c^Department of Earth and Planetary Sciences, Rutgers University, New Brunswick, NJ 08854; ^d^Department of Physics, Federal University of Santa Catarina, Florianopolis 88040-900, Brazil; ^e^Department of Plant Biology, Institute of Biology, University of Campinas, Campinas 13083-875, Brazil; ^f^School of Forest, Fisheries and Geomatics Sciences, University of Florida, Gainesville, FL 32611; ^g^Laboratório de Ecologia da Conservação Programa de Pós Graduação em Biodiversidade Universidade Federal do Oeste do Pará, Santarém 68037-110, Brazil; ^h^Departamento de Sistemática e Ecologia, Centro de Ciências Exatas e da Natureza, Universidade Federal da Paraíba, João Pessoa 58050-585, Brazil; ^i^School of Environment and Sustainability, University of Saskatchewan, Saskatoon S7N 5C8, Canada; ^j^North China University of Water Resources and Electric Power, Zhengzhou 450046, China; ^k^School of Geography, Earth and Environmental Sciences, University of Birmingham, Birmingham B15 2TT, United Kingdom

**Keywords:** transpiration source water, embolism resistance, Amazon, dry season, moisture recycling

## Abstract

Transpiration drives most of the local rainfall during the dry season in the Amazon forests by recycling moisture into the atmosphere. However, the source, temporal origin of transpiration, and spatial distribution of transpiration water sources remain unclear. Here, we quantify transpiration sources across a topographic gradient in the eastern Amazon. We show that on hills, dry-season transpiration sources are mostly shallow soil water recharged by dry-season rainfall. This is different in valleys, where tree water sources include both shallow and deep soil layers, with both dry- and wet-season contributions. We show that species embolism resistance largely explains this pattern in tree water use but with contrasting trade-offs between topographic positions. The significant relationship between embolism resistance and depth of water uptake in both hill and valley species may merit incorporation into process-based models to understand changes in vegetation and land surface fluxes.

## Significance

Transpiration sustains the water balance and climate of Amazon forests. During the dry season, it plays an even more critical role by supplying atmospheric moisture to produce rainfall. But explaining the sources of transpiration across different species and the landscape remains a long-standing challenge in ecohydrology. Here, we show that embolism resistance—a hydraulic trait measuring species drought resistance—strongly controls transpiration water sources across the landscape. In the dry season, a period of increased transpiration rates, sources on hills include dry-season rainfall from shallow soil layers. In valleys, sources also include older precipitation stored in deeper layers. Critically, embolism resistance controls transpiration age and can be used to parameterize vegetation water use in hydrologic and ecosystem models.

Transpiration is the largest terrestrial water flux on the global land surface (1). In the Amazon forest, Earth’s largest tropical forest, transpiration feeds “flying rivers” (2, 3) where at least 64% of all recycled moisture in the Amazon has traveled through the leaves of trees (4, 5). Transpiration contributes up to 70% of dry-season rainfall (4). This contribution of transpiration to rainfall is particularly important for more water-stressed forests in the eastern Amazon (i.e., the more seasonal Amazon), where transpiration contribution to atmospheric moisture can be larger (5–7). Dry-season transpiration is also key to triggering the onset of the wet-season rainfall (8). Early evidence of this was the delay in the wet-season onset following a severe evapotranspiration reduction in 2005 drought (9). Thus, in the more seasonal Amazon, transpiration is critical to sustaining the water balance and climatic conditions in the region (10–12). However, we lack a mechanistic understanding of the source of transpiration in these forest ecosystems (13). And an open research question is as follows: What water stored in the subsurface is returned to the atmosphere by vegetation during the dry season? This lack of mechanistic understanding of transpiration water sources hampers our ability to mechanistically represent vegetation response to drought in vegetation process models (14) and improve the parameterization of transpiration fluxes and the connectivity between surface and subsurface waters in Earth system models (15–17).

Dry-season transpiration in the more seasonal Amazon exceeds that observed during the wet season, with evapotranspiration rates (ET) surpassing precipitation inputs (18–20). To meet this increase in atmospheric demands of the dry season, forests likely access water stored in deeper soil layers (21–24). However, direct observations of the source of transpiration in the more seasonal Amazon are limited to extremely dry years, such as El Niño periods, and from hilltop areas (22, 23). Across various biomes, a notable shift to deeper water sources occurs when surface soil layers begin to dry (13), indicating a dynamic response to moisture availability that cannot solely be attributed to the distribution of fine roots (25, 26). The capacity of species to uptake water in drier soils and maintain transpiration is influenced by a complex interplay of soil properties, water availability, and specific hydraulic traits, such as resistance to xylem embolism and root hydraulic redistribution (27–30). The drier conditions in the Amazon are already pushing species to function beyond their hydraulic thresholds (31), which leads to reduced tree growth and increased mortality rates (32, 33). Consequently, this reduction in growth pushes the Amazon forests toward a state of carbon neutrality or pushes the forest into a carbon source rather than a sink state (34, 35). Therefore, developing a nuanced mechanistic understanding of the dry-season transpiration sources in the Amazon forests—particularly how these are connected to the hydraulic traits that sustain water extraction under drying conditions—is crucial for predicting how Amazon water and energy cycles will respond to projected increasing water stresses in the future (36–39).

Some studies in semiarid and Mediterranean climates have explored the links between transpiration water sources and stomatal conductance, leaf water-use efficiency, and CO_2_ assimilation (40–43), but results have been inconsistent on how water use strategies might impact source water uptake. Others have investigated the relationship between source water and leaf water potential (22, 44, 45), but these have not always explained transpiration water sources (46, 47). There is an overall lack of empirical evidence regarding the relationship between plant hydraulic sensitivity to drying soils and the sources of transpiration water across many biomes (13). Mechanistically, water moves from areas of higher potential in the soil to lower potential within the roots, requiring plants to maintain lower water potential than soils to access moisture. Despite sustaining lower water potentials, plants must maintain xylem conductance to support transpiration by preventing air entry in the xylem which causes blockages (i.e., embolism). Embolism resistance—the relationship between a leaf water potential and the loss of xylem conductivity—could play a crucial role in understanding how plants continue to uptake water and transpiration sources during drought. This balance underscores the interplay between hydraulic traits and plant functioning during drought. The water potential at which 50% of a plant’s xylem hydraulic conductivity is lost due to embolism is known as P50. The scarcity of empirical data on these dynamics between transpiration source water and hydraulic traits hinders our understanding of the sources of moisture recycling in the eastern Amazon via transpiration and limits the integration of these processes into predictive models of forest responses to drought. Great uncertainty exists regarding water source depths in the subsurface (i.e., deep vs shallow soil layer), the temporal origin of this water transpired source (i.e., wet-season or dry-season rainfall), and how transpiration sources might differ in different landscape positions (e.g., hills vs valleys) and across species with different hydraulic thresholds, i.e., more or less tolerant to xylem embolism.

Here, we report on the transpiration source water in the seasonal Amazon (5-mo dry season) across a topographic gradient during a normal year (i.e., without extreme high or low rainfall regime; see *SI Appendix*, Fig. S1). Our central questions are as follows: i) What is the source of transpiration in the seasonal Amazon? ii) What is the temporal origin of these sources (precipitation in the current dry season vs. previous wet season)? iii) How do transpiration depth and origin vary across topographic gradients and species with different embolism resistance growing under the same overall climatic conditions? We conducted our field campaign during the dry season in the Tapajós National Forest (eastern Amazon) across a topographic gradient at two sites: one on a hill and one at a river valley site (*SI Appendix*, Fig. S2). During this campaign, we traced transpiration sources by collecting samples from xylem, soil, groundwater, and streams as well as rainfall events that occurred during the sampling period for isotopic analysis of hydrogen and oxygen (δ^2^H and δ^18^O) as tracers of tree water sources. We leverage embolism resistance (P50) data collected in the same sites during this same field campaign (48). We then used a mixing model (49) to identify the source of transpiration in the topographic gradient and its relation to tree embolism resistance.

## Results and Discussion

### Transpiration Water Source From Hill to Valley.

**Fig. 1.**
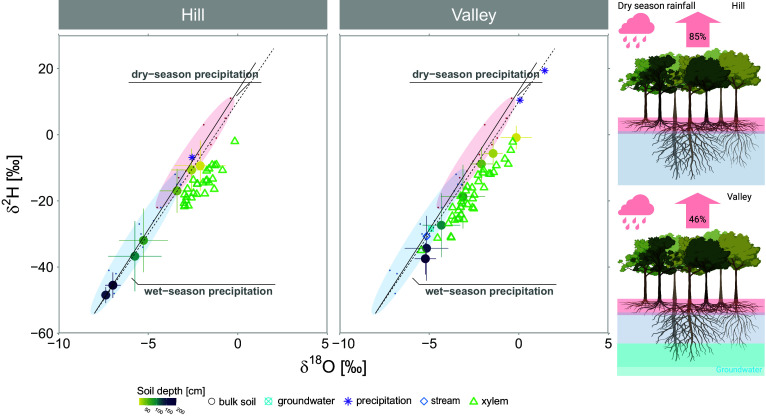
Dry-season transpiration source water in dual-isotope space. The first panel (*A*) shows the overlap between xylem water and shallow soil water (≤50 cm; 10, 25, 50 cm - yellow to light green circles), indicating the dominant use of this source on the hill. Soil circles show the mean soil water isotopic composition per soil depth colored by depth, and the SD (lines). The second panel (*B*) shows the overlap between xylem water and the entire soil water distribution in the valley (10 to 175 cm soil depth). Both panels show the dry- and wet-season precipitation distribution in dual-isotope space, where the dots are the monthly precipitation values (GNIP and Bowen; see Methods). The dashed line shows the GMWL (Global Meteoric Water Line), and the solid line is the LMWL (Local Meteoric Water Line). The third panel is our perceptual model (*C*) showing dry-season transpiration source water on the hill and in the valley without accounting for sampled species basal area. On the hill, shallow soil water recharged by dry-season precipitation contributes to 85% of transpiration, whereas this source contributes to 45% of the valley. The (*C*) panel was created in https://BioRender.com.

**Fig. 2.**
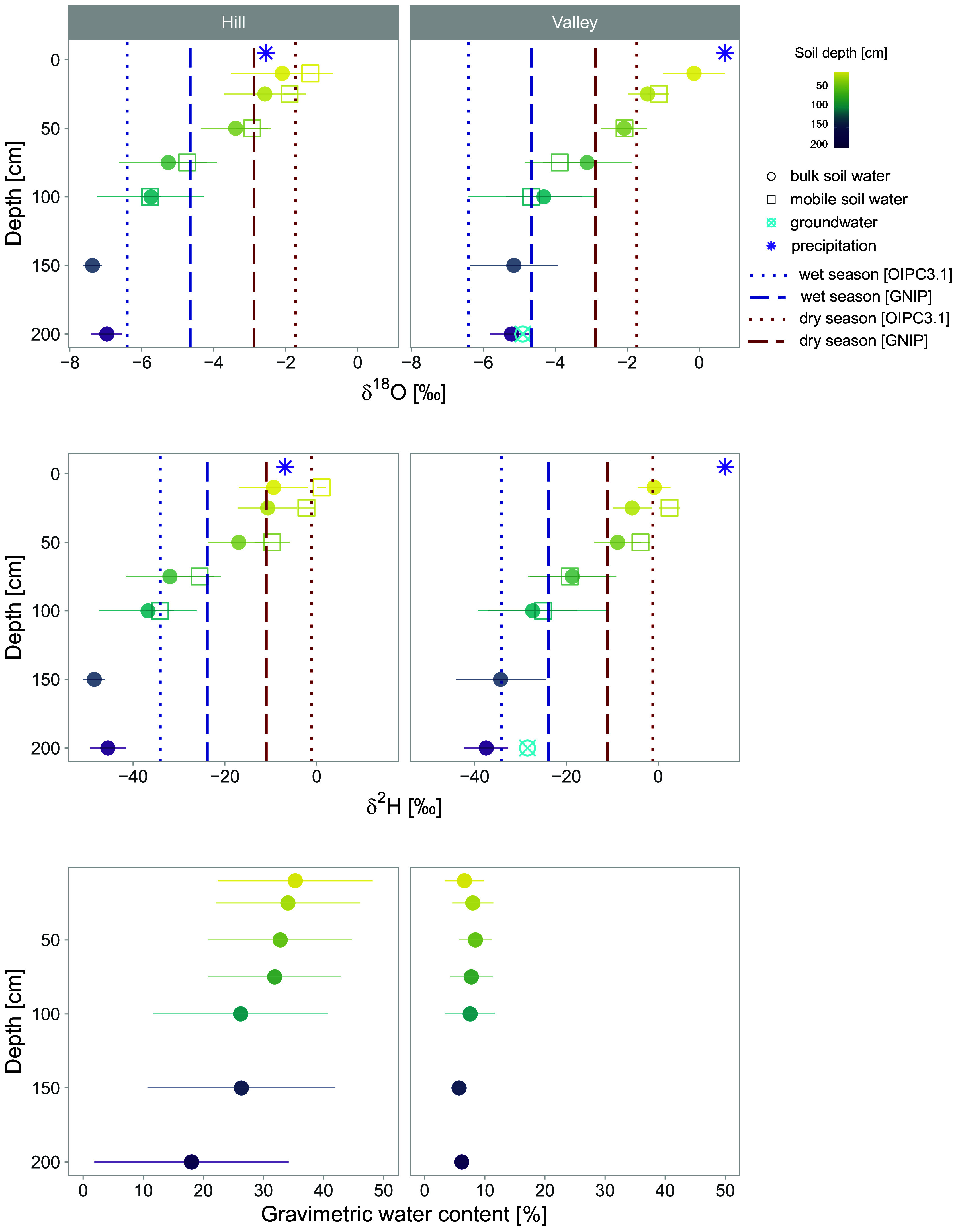
The isotopic composition of soil profile on the hill and in the valley. The upper plot shows δ^18^O values and lower plot δ^2^H values across the soil profile for both bulk soil and mobile soil water along with precipitation (i.e., throughfall) collected during sampling and groundwater. The seasonal precipitation isotopic signature is shown by dashed lines, OIPC3.1, precipitation volume corrected (52, 53) and GNIP, raw data (54). The average gravimetric water content of the samples is shown at the bottom.

We found that dry-season precipitation is the dominant source of transpiration on hills, while transpiration in the valley is sustained by both dry- and wet-season precipitation (Figs. 1 and 2). Shallow soil water (≤50 cm) reflected most dry-season precipitation isotopic signatures and was statistically equal to dry-season precipitation (*P* > 0.05) (Fig. 2). We also observed a larger gravimetric water content in shallower soil layers on the hill compared with deep layers (*P* < 0.05) in which water content progressively declined with depth (Fig. 2). The sandy soils from the valley do not hold large volumes of water compared to hill soils, but also showed slightly higher water content in shallower layers, although not significantly higher (*P* > 0.05). By the time the sampling took place between the end of September and early October, the region had already experienced 3 mo of dry season, during which ET significantly exceeds monthly precipitation inputs (18, 20). The high ET rates with reduced rainfall and greater gravimetric water content at the surface indicate the recharge of shallow soil layers by recent dry-season rainfall. This is further supported with the similarity in isotopic signatures between collected rainfall events and shallow soil water (*P* > 0.05). Additionally, shallow soil δ-values are distinct from wet-season rainfall in the valley (*P* < 0.001) and hill (*P* < 0.05). On the hill, while the shallow soil layers overlap with dry-season precipitation distribution, the 50 cm depth plots in a region where dry-season and wet-season rainfall distribution overlap in dual-isotope space plot (Fig. 1). The average seasonal origin index (SOI) (50) of the water in this layer was 0.35, which indicates that it was more influenced by dry-season rainfall (closer to 1) than wet-season (further from −1). While it supports the larger origin from the dry season, a greater spatial sampling resolution could have helped to elucidate the transition of influence between wet- and dry-season precipitation in the soil profile. Deeper soil layers (≥75 cm) exhibited isotopic values similar to the wet-season rainfall in both sites (*P* > 0.05) (Fig. 2). Previous hydrometric investigations in another seasonal site in the Amazon showed that soil moisture below 1 m is not influenced by precipitation during the dry season, and only wet-season inputs recharged the soil below this depth (51), supporting our isotopic observations (Fig. 2). In the valley, deep layers are also not significantly distinct from groundwater and stream isotopic signatures (*P* > 0.05). In contrast, stream and groundwater are distinct from shallow soil layers (*P* < 0.05).

Precipitation infiltrates the soil before being taken up by roots and feeding transpiration fluxes. On the hill, transpiration is fed by shallow soil water (in the upper 50 cm), mostly recharged by current dry-season precipitation, with xylem water overlapping the overall distribution in shallow soil layers. In contrast, valley trees water uptake is distributed throughout the soil profile, including deeper layers (Fig. 1). Xylem water isotopic signatures in both sites plot below the LWML but within the range of soil water distribution, indicating that xylem water relies, or partially relies, on soil water that has undergone evaporative fractionation in shallower layers. While uncertainties might exist in cryogenically extracted xylem water signatures (55, 56), we used high-temperature extraction protocols, which minimize potential deuterium offsets (57, 58). Additionally, cryogenically extracted xylem water is not always distinct from more passive and direct sampling approaches (e.g., in situ) (59, 60), and cryogenic deuterium bias seems to be associated with samples with low water volume (61), which was not our case (the extracted xylem volumes per sampled vial were >0.68 ml; the mean was 1.5 ml).

Computed overall source contribution to transpiration from the distinguished soil layers through mixing model (MixSIAR) analyses showed that, on the hill, shallow soil water (upper 50 cm soil depth) contributes to 85% (±6% SD) of transpiration sources during the dry season, whereas in the valley, the same source contributes to 45% (±0.5% SD) of tree water uptake (Fig. 1). This observed transpiration source water reflects the tree water use of the dominant species at each topographic location. At this highly diverse site, we sampled species covering 14% and 26% of the total basal area on the hill and in the valley, respectively. By adjusting the observed transpiration source water to the relative basal area of each sampled species, the overall shallow-dry season water use increases slightly to 46% in the valley and decreases to 69% on the hill. The large and statistically significant isotopic difference between the end members, deep and shallow layers, especially for deuterium on the hill (shallow δ^2^H mean: −12.34‰; CI: −15.12‰ and −9.55‰; deep δ^2^H mean: −37.45‰; C.I.: −41.58‰ and −33.31‰) and in the valley (shallow δ^2^H mean: −5.13‰; C.I.: −7.03‰ and −3.23‰; deep δ^2^H mean: −26.46‰; C.I.: −30.71‰ and −22.19‰) reduces uncertainties in source estimates that could be a result of potential cryogenic bias (62).

The isotopic observations of shallow, and therefore more predominant, dry-season water use corroborate with hydrometric observations of precipitation and transpiration. During the dry season, transpiration rates are reported to reach 3 to 4 mm/d (19, 63), totaling 90 to 120 mm/mo, which aligns closely with independent evapotranspiration measurements (18, 20). With the average monthly rainfall of approximately 60 mm during the dry season (*SI Appendix*, Fig. S1), rainfall can contribute to approximately 50 to 67% of the total transpiration demand. These hydrometric estimates provide a reasonable match with our isotopic findings, suggesting that about 46 to 69% of the water used for transpiration originates from dry-season precipitation and that trees maximize dry-season precipitation water use, albeit with inherent uncertainties due to variations in basal area and species-specific patterns in water usage.

Isotopic evidence robustly supports recent modeling findings, demonstrating that 60% of transpiration in the Tapajós National Forest during the dry season is sustained by the current month’s precipitation (64), with the remainder linked to the previous month’s events. Our study refines this understanding by using isotopes to explicitly attribute the previous month’s precipitation to the wet season specifically rather than more generally to earlier months (e.g., within the dry season). Additionally, our isotope tracing has uncovered the unique distribution of water sources across hill and valley positions in the Amazon forests. Valleys, which have shallow water tables (<5 m below the surface), make up 36% of the Amazon basin (65) and trees in the valley rely almost evenly on dry- and wet-season rainfall, whereas hills, which constitute the majority of the basin (65), relies mostly on the dry-season rainfall.

This reliance on dry-season precipitation underscores the importance of current-season moisture recycling for the Amazon’s climate, suggesting that reductions in transpiration could significantly impact the precipitation regime and disrupt moisture recycling cascading effects across the region (4). Previous studies have shown that transpiration is a major contributor to atmospheric moisture and local rainfall (66–69). This is even more critical during the dry season, where up to 70% of rainfall comes from transpiration (4). Our findings highlight that dry-season rainfall is the primary source of this transpiration in years of normal precipitation regimes (*SI Appendix*, Fig. S1), where trees potentially recycle their own rainfall. Thus, reduced dry-season precipitation can impact Amazon moisture regimes, where trees cannot access deeper water sources.

Our research supports foundational studies demonstrating the critical reliance of Amazon forests’ biomass and transpiration on dry-season rainfall. Prior throughfall exclusion experiments, including those that specifically excluded dry-season rainfall, have shown that a 50% reduction in rainfall significantly diminishes transpiration rates and results in a 20% loss in biomass over seven years (70, 71). Furthermore, during extremely dry periods, such as ENSO years, trees on hills may increasingly tap into deeper water layers (22, 72). However, the observed reductions in transpiration and biomass production (70, 71) raise concerns that deeper water sources may not consistently satisfy the water demands of these tropical forests, as shown in other tropical sites (24). This limitation could be due to the physical inaccessibility of deeper water resources (e.g., deeper roots), or even when roots are present (27, 73), plant hydraulic thresholds might limit their ability to withdraw water in drier soils held under low water potential gradients (28, 74). Our work sought to address these gaps by providing a mechanistic explanation of sources from a tree hydraulic perspective.

### Embolism Resistance Explains Transpiration Source Water.

**Fig. 3.**
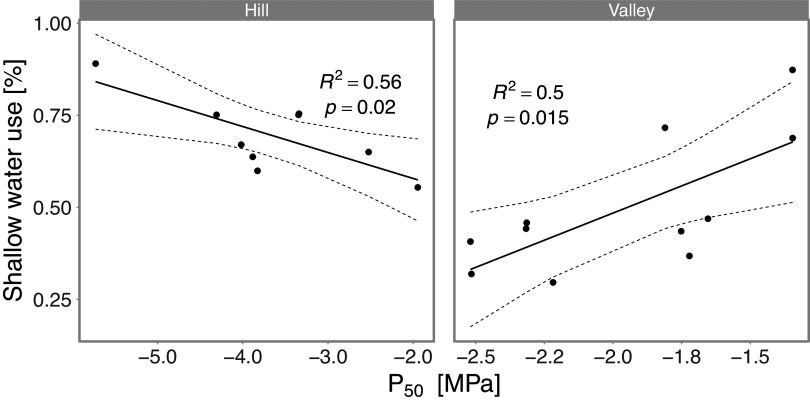
Relationship between shallow water contribution to transpiration and species embolism resistance on the hill and valley. Individual points indicate species mean shallow water use and embolism resistance. Shallow water use [%] closer to 1 denotes species with dominant uptake of shallow water (≤50 cm), and values lower than 0.5 denote species with dominant deep-water use (≥75 cm). The dashed lines represent the 95% bootstrapped CI for the slopes and intercepts. The R^2^ of each regression is displayed in the figure.

The mechanistic explanation for the larger contribution from shallow layers and current dry-season rainfall is directly linked to trees’ ability to withdraw water in drying soils while maintaining xylem hydraulic conductance—embolism resistance. Our data showed a strong and significant link between the critical plant water potential (P50) and the contribution of the shallow layer (Fig. 3), in both hill (R^2^ = 0.56; *P* = 0.02) and valley species (R^2^ = 0.50; *P* = 0.01). Embolism resistance explained overall 50% of total shallow water use (Fig. 3). This indicates an important alignment between hydraulic traits and patterns in tree water use and response to water availability in the seasonal Amazon. Embolism resistance has been related to important structural traits that directly or indirectly influence tree’s response to water availability and survival (22, 75). However, its direct influence on the distribution of transpiration source water has not yet been tested. Our data reveal that differences in water source partitioning between hills and valleys reflect distinct hydraulic strategies of trees at each site. In the hill, where trees predominantly utilize shallow water sources during the dry season, species exhibited higher embolism resistance (P50), allowing greater use of dry-season rainfall. Conversely, valley species that used shallow water sources showed greater vulnerability to embolism (Fig. 3), suggesting a reliance on deeper water sources among those with higher embolism resistance.

The opposite relationship between embolism resistance and tree water uptake pattern between hill and valley shows different tree hydraulic strategies for different topographic positions under the same rainfall regime. The hill species less resistant to embolism tended to show larger contributions from deeper sources (still within the first 2 m), aligned with the drought resistance-avoidance trade-off (76); wherein vulnerable species might develop other strategies to avoid drought stress (i.e., deeper roots, stomatal control). This trade-off, commonly observed across different topographic and canopy positions, underscores the vulnerability of species closer to the water table and the higher embolism resistance of those unable to access deeper resources (48, 77) or between trees in different canopy positions and distinct below-ground root distributions (22, 78). We also observed this pattern within the same canopy strata in the hills, driven by the interplay between tree ecophysiology and sporadic rainfall during the dry season (Fig. 3).

However, this drought resistance-avoidance trade-off is not observed in the valley (Fig. 3). Here, species with higher embolism resistance (more negative P50) use more stable deeper water sources (i.e., groundwater). We hypothesize that the investment in deeper roots and high embolism resistance in the valley is a response to fluctuating water table conditions, which often rise close to the surface during the wet season, exposing roots more frequently to waterlogging. In the valley, the water table rises closer to the surface during the wet season, with values ranging from 0 to 80 cm below the surface at 12.5 m away from the stream between the wet and dry seasons, respectively. The lack of oxygen in the root system during waterlogging can lead to reduced aquaporin activity that modulates root water transport and restricted soil water uptake, mirroring drought responses in terms of physiological stress and decreased xylem conductance (79–83). Waterlogging thus induces a response similar to drought (83), which could explain the higher resistance to embolism in valley species drawing from deeper water sources. This adaptation likely reflects frequent waterlogged conditions, emphasizing embolism resistance as a critical factor defining the ability of vegetation to utilize dry-season rainfall and influencing long-term water balance in ecosystems by affecting the mobilization of younger or older water storage. We did not find a direct relationship between shallow soil water use and species’ average distance to the stream, nor between P50 and distance to the stream (*SI Appendix*, Fig. S4).

**Fig. 4.**
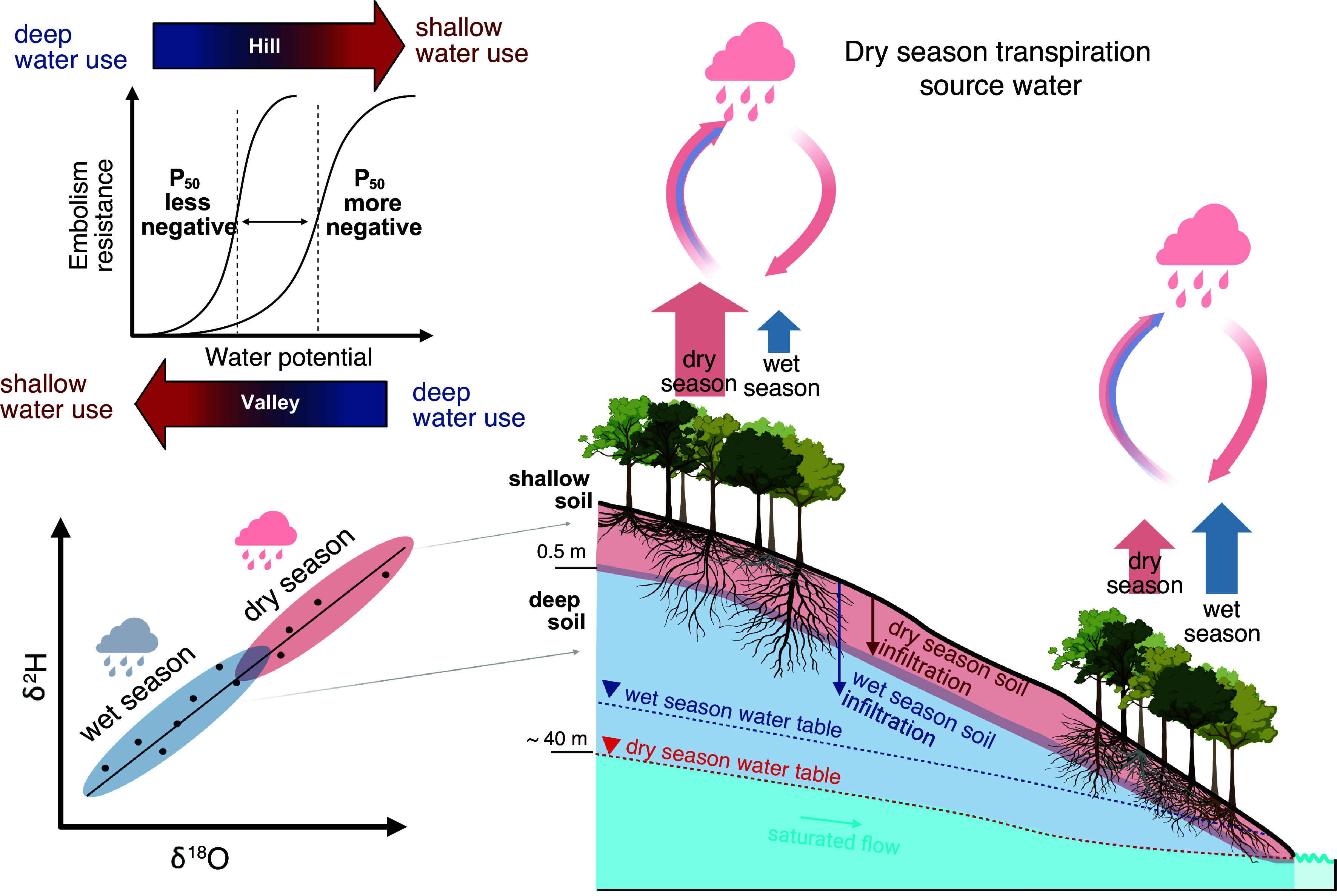
Transpiration sources water during the dry season in the eastern Amazon and its relationship to embolism resistance and topography position (hill and valley). Note that root distribution is attempting to approximate the reality, where more dimorphic roots (27), and variability of root depth (22) reaching greater depths have been described on hills (21), and shallower roots on valleys are expected given the proximity of the water table (85). This figure was created in https://BioRender.com.

We showed that transpiration source water in the more seasonal Amazon is driven by precipitation seasonality, embolism resistance, and topography, summarized in Fig. 4. The relatively equal contribution of shallow and deep soil water to transpiration in the valley during the dry season is also explained by embolism resistance, but the opposite relationship is observed on the hill. The dominant use of shallow soil water, and therefore, younger sources on the hill, is explained by the higher embolism resistance and recharge of shallow soil layers by dry-season precipitation in a period of increased transpiration rate. Recent catchment-scale water age modeling across the United States showed that the simulated mean water age of root water uptake is correlated with metrics of vegetation drought resilience derived from remote sensing (84). Similarly, they showed that in catchments where transpiration relies on younger water sources, vegetation experiences more frequent water limitation but is more resistant to drought, similar to hill species. In contrast, in catchments with vegetation featuring low resistance to drought, ET is mainly composed of older sources, similar to valley species (84). While our results support these findings, we show that species embolism resistance may be a stronger metric for predicting transpiration ages in an ecosystem and improving model parameterization.

### Implications for the Amazon Forests.

Overall, up to 69% of transpiration at our sites in the Amazon relies on current precipitation during the dry season, which is explained by tree embolism resistance. These observations have strong implications for moisture recycling in the Amazon (4, 86) as they indicate a relatively rapid turnover of dry-season rainfall that returns to the atmosphere through transpiration, a process distinct from that observed in other forest environments in temperate areas (87, 88). We showed that during the dry season, a period characterized by higher transpiration rates (18, 20), dry-season rainfall stored in shallow soil layers provides most of the water to supply transpiration in years of normal precipitation. Our results indicate that transpiration recycles most of the dry-season rainfall while also returning some older sources to the atmosphere (i.e., wet season). Our data additionally demonstrate that embolism resistance significantly influences transpiration age in this environment and contributes to the rapid return of recent precipitation to the atmosphere during the dry season. Importantly, transpiration is the dominant contributor to atmospheric vapor during this season in the seasonal Amazon over land (67, 68). Considering these factors, the increase in deforestation in the Amazon will, therefore, likely trigger reduced precipitation given this significant dependence on the forest to produce its own rain, particularly in regions where low-level moisture is efficiently rained out before mixing in the atmosphere during the dry season, as observed in the eastern Amazon (89). While the eastern Amazon primarily relies on moisture advection from the Atlantic Ocean, deforestation in the east could reduce rainfall in central and western Amazonia by diminishing recycled moisture that would otherwise be advected westward. Since dry-season transpiration also triggers wet-season rainfall (8, 9), this feedback might also be lag-affected, amplifying basin-wide impacts across different seasons. Additionally, the evidence of reduced biomass in the Amazon forests and the change to carbon neutrality and source (33, 34, 90) can be exacerbated on hills with reduced dry-season rainfall since shallow water use is an important source, and deeper layers might not fully offset drying shallower soils (24, 70, 71). Our findings suggest that understanding and predicting changes in moisture availability in the Amazon and vegetation drought response to changes in precipitation regimes will be best accomplished by incorporating key tree hydraulic traits, such as embolism resistance, along topographic gradients. These results provide valuable empirical constraints for improving modeling efforts (e.g., refs. 17, 84), particularly by highlighting the role of embolism resistance in influencing transpiration sources and ages, and moisture recycling sources. To advance this understanding, targeted field measurements of this key hydraulic trait across topographic gradients are essential, as current observations of hydraulic traits remain limited (31). Such efforts will reduce uncertainties and enable more accurate model representations of transpiration sources and ages, and drought responses.

## Materials and Methods

### Site and Species Composition.

We conducted our investigation across a topographical gradient at the Tapajós National Forest, near Santarém, Brazil (3°51′S, 54°58′W) (*SI Appendix*, Fig. S2). The mean annual precipitation for the region is 2,212 mm (station: 25400, Santarém, Brazil, National Water and Sanitation Agency (ANA); *SI Appendix*, Fig. S1). Mean annual temperature and humidity are 25 °C and 85%, respectively (91). Here, we leverage two permanent research plots established by the Biodiversity Research Program (PPBio Santarém/POPA LTER) located within a large hillslope to conduct our tree water source investigation. The plot in the valley is ~100 m a.s.l. while the hill site (top of this gradient) is ~250 a.s.l. The water table depth at the valley site varies because this is also located on a small hillslope, ranging from 0 to 0.16 m, and from 0 to 0.8 m at 12.5 m away from the stream, between wet and dry season at 1 m away from the stream. The sampled individuals were located between 6 to 37 m away from the stream, but when grouped per species, the average distance was 14 to 27 m.

We sampled eight species on the hill and eleven in the valley for isotopic analysis. Those comprise the most abundant species in each location, but we also have co-occurring species or genera (*SI Appendix*, Table S1). All the tree hydraulic measurements and isotope sampling were conducted between the end of September and the beginning of October 2021.

### Embolism Resistance Measurements.

We used species mean P50 as an indicator of species embolism resistance (75, 77). The P50 is the xylem water potential at which 50% of loss of hydraulic conductivity occurs. We used the pneumatic approach to build the hydraulic vulnerability curves, the relationship between xylem water potential and percentage loss of xylem conductivity, and calculate the branch-level P50 and then species-level P50. A more detailed description of the method and data used in this study can be found in ref. 48. Embolism data is available at ref. 92.

### Field Sampling for δ^2^H and δ^18^O Analysis.

We collected canopy-level suberized branches for isotope sampling of xylem water in both hillslope positions. We collected canopy-level branches with the assistance of experienced local tree climbers. Immediately after the tree climber removed the branch from a targeted tree, we sampled the xylem of each branch by first removing the bark, then quickly chopping the wood and storing it in a glass vial (737 W Labco, UK). We collected at least two vials per branch (subsamples) and sampled 21 species (*SI Appendix*, Table S1), collecting, on average, two trees per species (individuals, n = 41) and sampling the same individual on more than one occasion during the sampling campaign. We collected bulk soil samples in three locations in each site using a soil auger. Samples were collected at 10, 25, 50, 75, 100, 150, and 200 cm depth and three subsamples per depth. Part of the samples were stored in glass vials (737 W Labco, UK) for cryogenic extraction, and the other portion was double sealed in coffee bags (Uline) for mechanical squeezing sampling. The first provides the bulk soil water isotopic composition, whereas the latter provides the more mobile soil water signatures that reflect more recent inputs (93). Besides plant and soil samples, we also collected a stream sample at the base of the hillslope and a groundwater sample from a well installed in the valley. Precipitation (i.e., throughfall) was collected at each site using three collectors distributed near sampling trees. Precipitation was collected on three occasions during the sampling campaign. We sealed all the vials with parafilm in the field and, on the same day, refrigerated all the samples. At the end of the field campaign, samples were transported in coolers to the Hillslope Hydrology Laboratory, University of Saskatchewan, where we extracted water from xylem and soil samples and conducted isotopic analysis of all samples.

### Sample Water Extraction and Laboratory Analysis.

For isotopic analysis and cryogenic extraction, we followed the protocol described in detail in ref. 94. Briefly, stream, groundwater, and precipitation samples were analyzed using a liquid water off-axis integrated-cavity output spectroscopy analyzer (IWA-45EP OA-ICOS; Los Gatos Research Inc., San Jose, CA, USA) with repeatability of 0.2 and 1.0 ‰ δ^18^O and δ^2^H, respectively. We cryogenically extracted soil and xylem samples. We used 180 °C for extraction and 15 and 24 min extraction time for soils and xylem, respectively. All subsamples were checked for a 98% extraction efficiency, and the samples that did not reach it were discarded. We determined the gravimetric water content of soil samples based on wet and dry weight after cryogenic extraction and oven drying. The squeezed soil samples followed protocols described in ref. 93. All soil-extracted water was also analyzed using an IWA-45EP OA-ICOS. Xylem water isotope analyses were carried out at the National Hydrology Research Centre Stable Isotope Laboratory using an Isoprime isotope ratio mass spectrometer (IRMS). Subsamples were analyzed individually, and only then averaged. Isotope data is available at ref. 92.

### Isotope Data From Long-Term Precipitation.

Because of the lack of long-term precipitation isotope or monthly isotope data at the studied site (Tapajós National Forest), we used the precipitation data from Global Network of Isotopes in Precipitation (GNIP), from the nearest station, which was located in Santarém (75 Km) (IAEA/WMO, 2024) (95) (*SI Appendix*, Fig. S1). We built the Local Meteoric Water Line based on the historical (1972–1973) monthly data from this station. The local meteoric water line slope is 8.41, and intercept is 13.38. Additionally, we used modeled mean-weighted monthly precipitation data (http://waterisotopes.org) (52) to compare against historical data and provide the mean-weighted seasonal precipitation value.

### Data and Statistical Analysis.

We used the δ^18^O and δ^2^H values of xylem, bulk soil water, wet-season, and dry-season mean weighted rainfall isotope data to assess the source of transpiration in seasonal Amazon. First, we used the Shapiro–Wilk test and histograms to evaluate normality assumptions in the isotope data. Given that most of the data were not normally distributed, we used nonparametric Kruskal–Wallis and subsequent post hoc Dunn test to assess the isotopic differences between available water sources. We assessed whether the isotopic compositions of shallow (<50 cm) and deep (≥75 cm) soil water were significantly distinct. Those layers were first determined based on the plotting distribution of each depth in dual-isotope space and across the soil profile. We used the same test to evaluate the difference in isotopic composition between shallow, deep, groundwater and stream, dry-season precipitation and wet-season precipitation. We grouped groundwater and stream during statistical analysis given the small variability in isotopic composition and that stream is baseflow (i.e., groundwater) during this period. Thus, we used them as the same source during statistical analysis. We adjusted *P*-values according to Benjamini and Hochberg to control false discovery rates. The significance level for all statistical tests was set to a 95% CI.

We used dual-isotope inference to compare the isotopic composition between xylem, soil, precipitation, and wet- and dry-season precipitation and identify tree water sources. We then used the MiXSIAR mixing model, a Bayesian framework with Markov chain Monte Carlo (MCMC) built on R (R Core Development Team, 2024) to quantify the source of transpiration, per site and per species. We used shallow (<50 cm) and deep (≥75 cm) soil water δ^18^O and δ^2^H as sources. We used those two layers because they plotted in different areas in dual-isotope space; additionally, shallow and deep δ^18^O and δ^2^H values were statistically distinct (*P* < 0.0001) in both hill and valley. In the valley, groundwater was not considered as an independent water source because it was not statistically distinct from deep soil water δ^18^O values (*P* = 0.416) and δ^2^H values (*P* = 0.491). The distinction of sources is a basic premise when using mixing models (49, 96). We ran one analysis for the valley and another for the hill, using δ^18^O and δ^2^H values from shallow and deep layers as sources and xylem δ^18^O and δ^2^H values. We used the raw data instead of providing the means and SD. The MCMC iterations defined by the parameter run length was selected as “long” for convergence, and the Gelman and Geweke diagnostics were acceptable (49). The discrimination values of δ^18^O and δ^2^H were set to 0 and no prior information was set in the model. Then, we grouped the xylem water from individuals of the same species per site and ran the same analysis to quantify transpiration source water per species. Details about MixSIAR model (v3.1) can be found in ref. 97.

We compared species’ embolism resistance (P50; in MPa) against the proportional use of shallow water obtained by the MixSIAR model to understand the relationship between trees’ embolism resistance and transpiration source water. We did this by fitting linear models with the “lm” function of the “nlme” package in R.

We used the SOI (50, 87) to investigate the origin of the water stored in the shallow soil layer (50 cm) that did not show deviations from the local meteoric water line (LMWL). Since the SOI method requires the projection of water back to the LMWL, which relies on temporal data from relative humidity and temperature at the site, which we lack, and given that large uncertainties can result from this calculation (98), we only applied the method to data already on the line. We used the weighted-mean precipitation from the dry and wet seasons as sources.

## Supplementary Material

Appendix 01 (PDF)

## Data Availability

Isotope data, embolism resistance data have been deposited in Zenodo (https://doi.org/10.5281/zenodo.15635631) (92). All study data are included in the article and/or *SI Appendix*. Previously published data were used for this work (https://nucleus.iaea.org/wiser) (95).
