# Supplementary material for: Embolism resistance supports the contribution of dry-season precipitation to transpiration in eastern Amazon forests
Source: Proc Natl Acad Sci U S A. 2025 Aug 14;122(33):e2501585122. doi: 10.1073/pnas.2501585122 (PMC12377737; doi:10.1073/pnas.2501585122)
Supplement: Supplementary file 1 — Appendix 01 (PDF) [file pnas.2501585122.sapp.pdf]

PNAS

Supporting Information for

Embolism resistance explains the dominant contribution of dry-season precipitation to transpiration in eastern Amazon forests

Magali F. Nehemy*, Caio R. C. Mattos, Rafael S. Oliveira, Marina Hirota, Ying Fan, Monique B. Schlickmann, Deliane Penha, Leandro Giacomin, Julliene S. G. M. Silva, Mayda Rocha, Gleicy A. Rodrigues, Jeffrey J. McDonnell

Magali F. Nehemy
Email: magali.nehemy@ubc.ca

This PDF file includes:

Supporting text
Figures S1 to S4
Tables S1

Supporting Information Text

Historical information on precipitation seasonality and isotopic composition. The long-term nearby precipitation data (station: 25400, Santarém, Brazil, National Water and Sanitation Agency (ANA)) shows that the total annual precipitation is 2212 mm (1991-2021), with an average dry season precipitation of 307 mm and 1905 mm during the wet season. In 2021 the total annual precipitation was 2422 mm. The larger rainfall above the long-term mean is a result of larger precipitation in November and December (Fig. S1). Therefore, this increase in precipitation above normal conditions occurred after the sampling period. The total accumulated dry season precipitation until October 2021 (sampling period) was 296 mm, which corresponds to the standardized anomaly of only 0.52, allowing us to characterize it as a normal dry season.

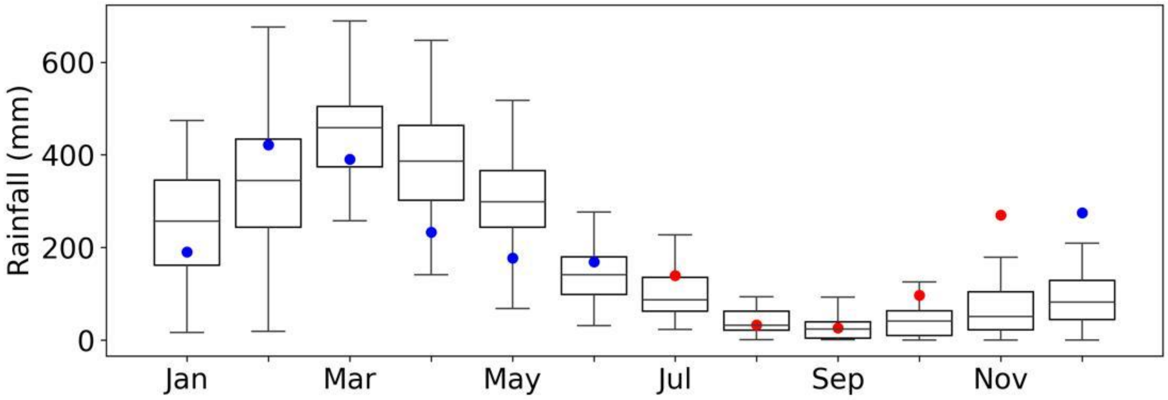

Fig. S1. Monthly precipitation from 1991 to 2021 period near the studied site (station: 25400, Santarém, Brazil, National Water and Sanitation Agency (ANA). Dots show sampling year monthly precipitation values; blue indicates the wet season (December to June), and red indicates the dry season (July to November). We conducted field sampling at the end of September and early October 2021.

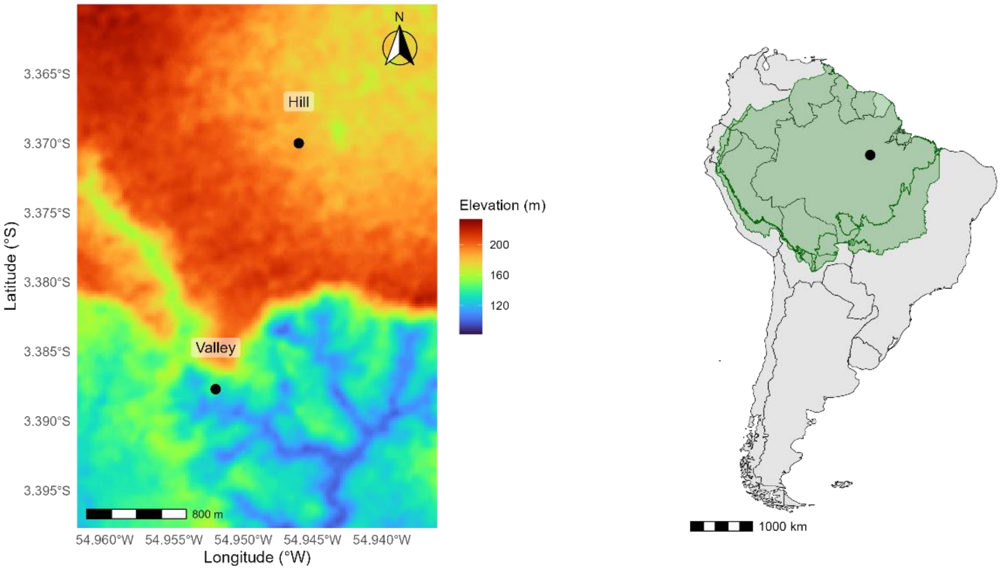

Fig. S2. Location and topographic variation of the two study site locations, hill and valley, within the Tapajós National Forest, Amazon. The detailed DEM map shows the hill and valley sites overlaid on elevation data derived from NASA SRTM (1). The inset map provides regional context, showing the study area (black dot) within the Amazon biome boundaries (in green) obtained from RAISG (2) and South America. Country boundaries are from Natural Earth (3). Map visualization was produced in R using the tidyverse (4), sf (5), terra (6), elevatr (7), cowplot (8), rnaturalearth (9), and viridis (10) packages.

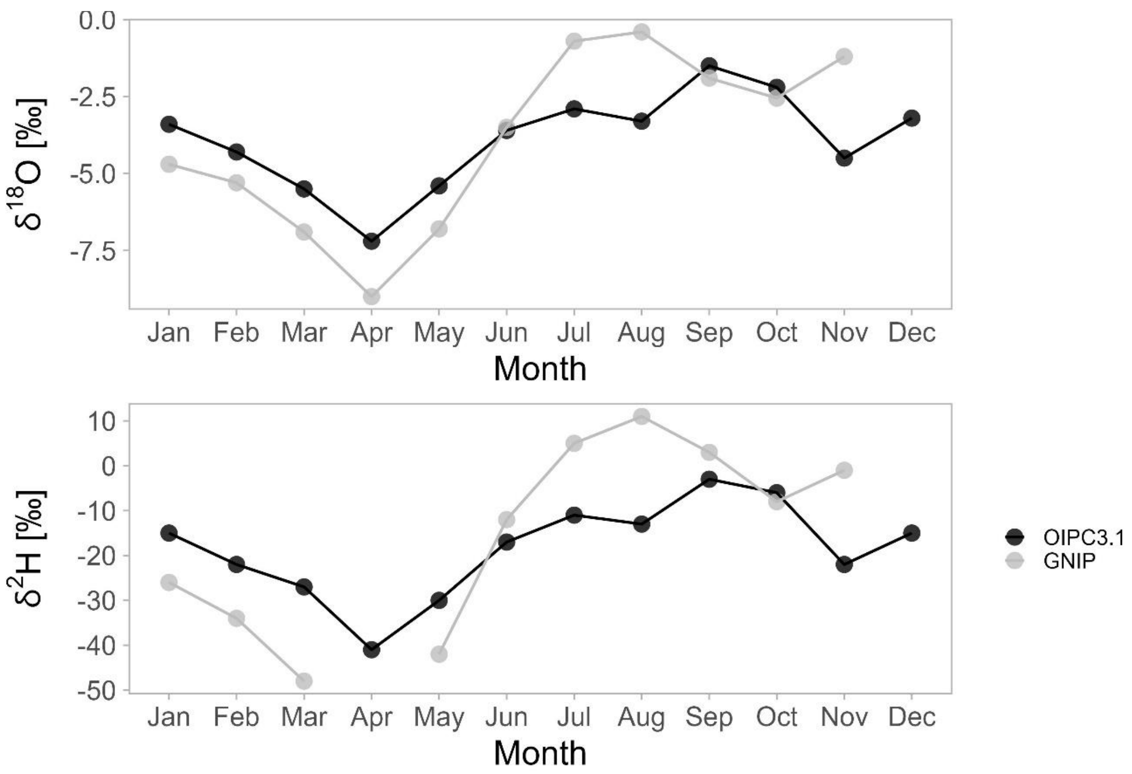

Fig. S3. Annual monthly precipitation isotopic values. 'GNIP' shows historical data and 'OIPC3.1' shows modeled values (https://wateriso.utah.edu/waterisotopes/) (See Material and Methods).

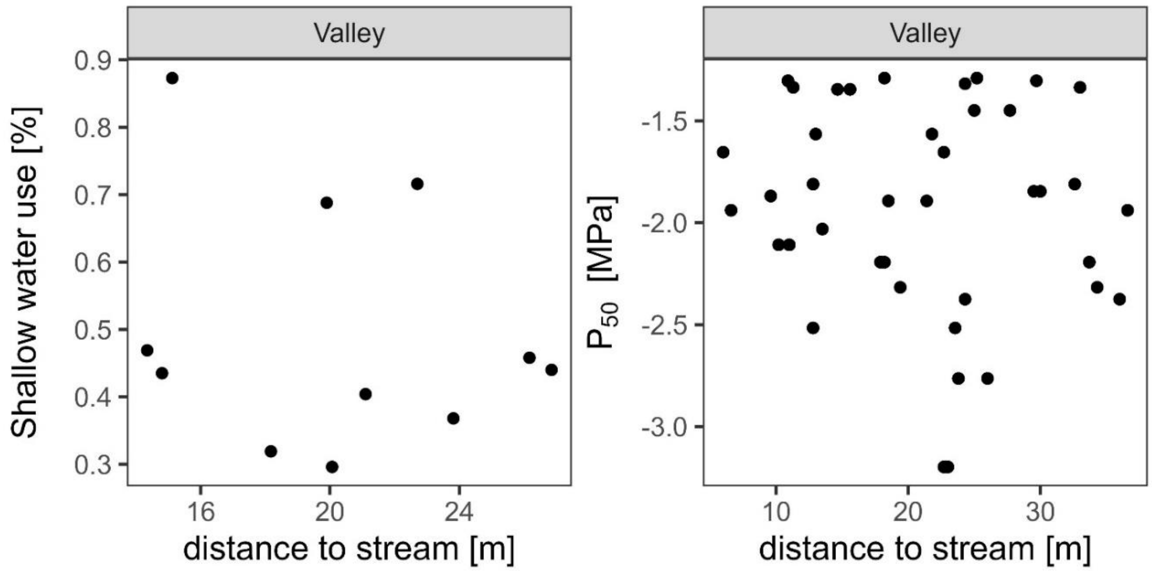

Fig. S4. Lack of relationship with stream distance. Lack of relationship between percentage of shallow water use and species mean distance to stream (first panel) and between species P_{50} and individuals' distance to stream (second panel).

Table S1. Species list per site (hillslope and valley) that were both sampled for isotopic analysis and P_{50} measurements.

**Species (Family)**	**Site**	**Species basal area per site (m^2)**
Chamaecrista scleroxylon (Fabaceae)	Valley	3.78
Chamaecrista scleroxylon (Fabaceae)	Hillslope	8.21
Chrysophyllum amazonicum (Sapotaceae)	Valley	1.15
Coussarea paniculata (Rubiaceae)	Hillslope	0.27
Eschweilera amazonica (Lecythidaceae)	Valley	1.74
Eschweilera apiculata (Lecythidaceae)	Valley	10.01
Eschweilera bracteosa (Lecythidaceae)	Valley	0.39
Eschweilera cf. amazonica (Lecythidaceae)	Hillslope	1.13
Eschweilera cf. micrantha (Lecythidaceae)	Valley	3.43
Eschweilera collina (Lecythidaceae)	Hillslope	0.08
Eschweilera coriacea (Lecythidaceae)	Valley	7.50
Eschweilera grandiflora (Lecythidaceae)	Hillslope	1.74
Eschweilera obversa (Lecythidaceae)	Hillslope	1.70
Eschweilera pedicellata (Lecythidaceae)	Valley	1.47
Minquartia guianensis (Coulaceae)	Valley	0.46
Minquartia guianensis (Coulaceae)	Hillslope	0.44
Brosimum sp. (Moraceae)	Valley	0.42
Pouteria bangii (Sapotaceae)	Hillslope	0.20
Pouteria cf. baehniana (Sapotaceae)	Valley	0.71
Pouteria cf. reticulata (Sapotaceae)	Hillslope	0.56

References

1. NASA Jet Propulsion Laboratory. NASA Shuttle Radar Topography Mission Global 1 arc second. 2013. NASA EOSDIS Land Processes DAAC. Available at: https://doi.org/10.5067/MEaSUREs/SRTM/SRTMGL1.003
2. Amazon Network of Georeferenced Socio-Environmental Information (RAISG). Amazonia Under Pressure Maps. 2022. Available at: https://www.raisg.org/en/maps/
3. Natural Earth. Free Vector and Raster Map Data. 2023. Available at: https://www.naturalearthdata.com
4. Wickham H, et al. Welcome to the tidyverse. *J Open Source Softw.* 2019;4(43):1686. https://doi.org/10.21105/joss.01686
5. Pebesma E. Simple Features for R: Standardized Support for Spatial Vector Data. *R J.* 2018;10(1):439-446.
6. Hijmans RJ. *terra: Spatial Data Analysis*. R package version 1.7-65. 2023. Available at: https://cran.r-project.org/package=terra
7. Hollister J, Shah T. *elevatr: Access Elevation Data from Various APIs*. R package version 0.4.3. 2023. Available at: https://cran.r-project.org/package=elevatr
8. Wilke CO. *cowplot: Streamlined Plot Theme and Plot Annotations for ggplot2*. R package version 1.1.1. 2020.
9. South A. *rnaturalearth: World Map Data from Natural Earth*. R package version 0.1.0. 2017. Available at: https://cran.r-project.org/package=rnaturalearth
10. Garnier S, et al. *viridis - Colorblind-Friendly Color Maps for R*. R package version 0.6.2. 2021.
